# Effects of modified atmosphere packaging on the postharvest quality of mulberry leaf vegetable

**DOI:** 10.1038/s41598-022-15257-9

**Published:** 2022-06-28

**Authors:** Teng-da Yang, Yu-long Chen, Fan-kun Zeng, Ming-qiang Ye, Ling Wang, Zheng Luo, Ying-wei Qi, Fei-ping Chen

**Affiliations:** 1 Sericultural and Agri-Food Research Institute Guangdong Academy of Agricultural Sciences/Key Laboratory of Functional Foods, Ministry of Agriculture and Rural Affairs/Guangdong Key Laboratory of Agricultural Products Processing, Guangzhou, 510610 People’s Republic of China; 2 College of Food Science of Southwestern University, Chongqing, 4007151 People’s Republic of China

**Keywords:** Biochemistry, Plant sciences, Engineering

## Abstract

Fresh mulberry leaf vegetable is nutritive and becoming popular. However, available preservation technologies are deficient. In present work, the effects of two kinds of modified atmosphere packaging on postharvest quality of fresh mulberry leaf vegetable stored at 4 °C were evaluated. The respiration rate of samples in the modified polyethylene packages (MP20) was 12.88–22.65% lower than that in normal polyethylene packaging (CK). The content of total soluble solids, soluble protein, and total polyphenol in MP20 was less changed than that in CK, and the vitamin C retention was higher as well. Moreover, the lignin content in MP20 was lower than that in CK during storage (19.79% vs 13.38% at day 8), and that was significantly positively related to the polyphenol oxidase and peroxidase activities inhibition. Taken together, a packaging with moderate gas permeability (MP20) is suitable for nutrition maintenance and lignification inhibition of fresh mulberry leaf vegetable during cold storage.

## Introduction

Mulberry leaf vegetable (*Morus alba* L.), consisting of a bud and two leaves at the top of the mulberry branch, are popular in some countries such as China, Turkey, Japan, and South Korea^1^. Modern pharmacological experiments have confirmed that mulberry leaves have multifunctional properties, such as anti-obesity^2^, antibacterial^3^, antioxidant^4^, and antidiabetic^5^. There are various kinds of products on the market, such as, semi-dry, quick-frozen, and fresh mulberry leaf vegetable. Compared with processed products, fresh mulberry leaf vegetable is more popular because of its fresh sweet taste. However, dehydration, etiolation, and lignification can easily occur in fresh mulberry leaf vegetable after harvest, which seriously affects its freshness and market value. Moreover, available preservation technologies for mulberry leaf vegetable are inadequate.

Packaging is important for quality keeping of vegetables during storage and selling. Among them, modified atmosphere packaging (MAP), with decreased O_2_ concentration and increased CO_2_ concentration with respect to normal air, is an effective technology for delaying vegetables deterioration after harvest^6^. For example, MAP could extend the storage time (at 25 °C) of Chinese cabbage from 2 to 5 days^7^, and reduce nutritional quality loss of pinemushrooms^8^. Matar et al. reported that MAP packaging could be a valuable option compared to standard packaging strategies especially at refrigeration temperature^9^. However, the application of MAP to fresh mulberry leaf vegetable during postharvest storage has not yet been reported.

Thus, the objective of the present work was to develop a MAP technology for fresh mulberry leaf vegetable preservation. Thus, two kind of packaging material with different gas permeability were chose to elucidate its effects on postharvest physiology (changes in headspace gas, respiration rate, relative conductivity and lignin), nutritional quality (vitamin C, total phenols, total soluble solids), and related enzyme activities (polyphenol oxidase and peroxidase) of mulberry leaf vegetable.

## Materials and methods

### Materials and sample preparation

Fresh mulberry leaf vegetable (obtained from mulberry trees directly) were purchased from Wan-zai sericulture professional cooperative, Qingyuan City, Guangdong Province, China, and transported to Guangdong Key Laboratory of Agricultural Products Processing within 3 h. Fresh mulberry leaf vegetable samples were randomly divided into two groups after precooling 20 min in a refrigerating house. Samples (200 g) were placed in a modified polyethylene bag (MP20) or a normal polyethylene bag (CK). The thickness of two bags was both 20 μm, while the O_2_ permeability of MP20 and CK was 8259 cm^3^ m^−2^ d^−1^ (0.1 Mpa)^−1^ and 20,083 cm^3^ m^−2^ d^−1^ (0.1 Mpa)^−1^, respectively, and CO_2_ permeability of both packages were about twice higher of the O_2_ permeability. All packages were stored at 4 °C and a relative humidity of 70%, and three packages of each treatment were taken at an interval of 2 days during 8 days storage for further evaluation. The gas compositions, respiration rate, soluble protein, total soluble solids, and relative conductivity were evaluated at the time of sampling, while the total polyphenol, vitamin C, lignin and enzymatic activities were analyzed after the samples were treated with liquid nitrogen and broken into powder.

### Gas composition analysis in the packages

The O_2_ and CO_2_ contents inside the packages were measured using a portable gas analyzer (Checkpoint II, PBI Dansensor, Ringsted, Denmark). Gas samples were obtained using a silicone septum affixed outside the packages. The results were expressed as percentages of O_2_ and CO_2_.

### Respiration rate

The respiration rate of fresh mulberry leaf vegetable was measured according to the method described by Chen et al.^10^. Mulberry leaf vegetable (100 g) in each bag were removed and put in a 3200 mL glass chamber with a CO_2_ analyzer (TN375, Taiwan). As soon as the chamber was sealed, the CO_2_ content inside glass chamber was recorded from 1 to 6 min. The respiration rate was expressed as mg CO_2_/kg^−1^ h^−1^.

### Soluble protein, total soluble solids, total polyphenol, and vitamin C content

The soluble protein content was determined using the Bradford method according to Wang et al. with some modification^11^. Briefly, mulberry leaves were mixed with deionized water in 1:1 (w/w), and grinded to get juice firstly. Then 0.5 mL of fresh mulberry leaf vegetable juice was added to 2.5 mL of Bradford solution. After incubation for 20 min, the absorbance was measured using a UV–Vis spectrophotometer (UV-1900i UV–Vis spectrophotometer, Shimadzu, Japan) at 595 nm. The soluble protein content was determined by an established standard curve, and the results were expressed as g/kg.

Total soluble solids (TSS) was measured according to the method of Mu et al.^12^. Five grams of mulberry leaf vegetable was filtered with cheesecloth and the filtrate was placed in a refractometer (RFM340+, Bellingham Stanley, Britain) for determination. The result was expressed as %.

Total polyphenol content was measured according to the method described by Scattino et al.^13^. Mulberry leaf vegetable powder (0.2 g) was extracted three times with 8 mL of 70% ethanol in an orbital shaker (DY-200B Shaker incubator, Tianjin, China) for 2 h. Then, the extract was centrifuged for 5 min at 3250*g* at 4 °C (Centrifuge 5810R, Eppendorf, Hamburg, Germany). The supernatant was diluted 10- fold with 70% ethanol, and then the diluent (1 mL) was mixed with 0.5 mL of Folin–Ciocalteu phenol reagent and 1.5 mL of 1 M sodium carbonate. The mixture was then incubated for 2 h at 25 °C. The absorbance was measured at 760 nm using a UV–Vis spectrophotometer. The total polyphenol content of mulberry leaf vegetable was determined using an established standard curve of gallic acid (R^2^ = 0.999), and expressed as mg of gallic acid per g of mulberry leaf vegetable on a fresh weight (FW) basis.

The vitamin C content was measured by fluorescence method, using a Cary Eclipse fluorescence spectrophotometer (Varian Inc., Palo Alto, CA, USA) according to Chen et al.^10^. The results were expressed as mg of vitamin C per g of mulberry leaf vegetable on a FW basis.

### Lignin content

Lignin content was measured according to the method described by Huang et al.^14^. Powder samples (0.5 g) were extracted for 10 min at 100 °C with 10 mL of 95% ethanol in an orbital shaker. The extract was centrifuged at 3250 *g* for 10 min, and the precipitates were washed with 95% ethanol for three times. The dried precipitates were dissolved in 1 mL acetyl bromide-ice acetic acid solution (25%, v/v), and the reaction was incubated for 30 min at 70 °C. The reaction was stopped by adding 1 mL of 1 M NaOH and 0.1 mL of 7.5 M hydroxylamine–HCl–acetic acid. After diluting 100 times, the absorbance of the supernatants was measured at 280 nm. Results were expressed as g per 100 g of mulberry leaf vegetable on a FW basis.

### Relative conductivity

Relative conductivity was measured using a conductivity meter (DDS-307A, Shanghai, China). Fresh mulberry leaf vegetable (0.2 g) were added to 20 mL deionized (ultra-pure) water and incubation for 20 min prior to the initial conductivity measurement (L_0_). The samples were then heated in boiling water for 20 min and cooled to room temperature prior to measuring their final conductivity (L_1_). The relative conductivity was determined using the following equation and expressed as percentage.

$${\text{Relative conductivity }} ({\text{\% }}) = \frac{{L_{0} }}{{L_{1} }} \times 100$$

### Activities of phenolic metabolism-related enzymes

Phenolic metabolism-related enzyme (Polyphenol oxidase (PPO) and peroxidase (POD)) were closely correlated with lignification of plant tissues. PPO activity was measured according to the method of Sukhonthara, Kaewka, and Theerakulkait^15^. Briefly, 0.6 g powder samples were extracted for 10 min with 2.4 mL of 50 mM phosphate buffer solution (PBS, pH 7.0) at 4 °C. The extract solution was centrifuged at 15,000* g* for 15 min at 4 °C. The supernatant was collected and diluted with PBS for 30 times before PPO activity determination. PPO activity was measured using catechol as a substrate according to a spectrophotometric method. The enzyme extract (0.1 mL) was rapidly added to 2.9 mL of 0.2 mol/L catechol solution prepared in PBS. The increase in absorbance at 400 nm was recorded for 3 min at 25 °C. One unit of PPO activity was defined as the amount of enzyme that caused a change of 0.01 in absorbance per minute per gram of sample tissue.

POD activity was measured according to the method of Huang et al.^14^, with a few modifications. Powder samples (0.4 g) were extracted for 10 min with 2.6 mL of 50 mM phosphate buffer solution (PBS, pH 7.0) at 4 °C. The extract solution was centrifuged at 15,000 *g* for 15 min at 4 °C, and the supernatant was collected for POD activity determination. The enzyme extract (0.05 mL) was rapidly added to 2.95 mL of substrate solution (2.55 mL of PBS + 0.2 mL of 4% guaiacol solution + 0.2 mL of 0.46% hydrogen peroxide) and mixed well in a vortexes mixer. The increase in absorbance at 470 nm was recorded for 3 min at 25 °C. One unit of PPO activity was defined as the amount of the enzyme that caused a change of 0.01 in absorbance per minute per gram of sample tissue.

### Statistical analysis

All triplicate data were analyzed by Fisher’s least significant differences (*P* < 0.05) of one-way ANOVA using SPSS software (version 22.0; SPSS Inc., Chicago, IL, USA).

### Ethics approval and consent to participate

Fresh mulberry leaf vegetable was purchased from Wan-zai sericulture professional cooperative, Qingyuan City, Guangdong Province, China. In this study, the experimental research, including collection of plant material, complied with relevant institutional, national, and international guidelines and legislation.

## Results and discussion

### Headspace gas composition

An atmosphere of 2–5% O_2_ and 3–10% CO_2_ in MAP has been used to extend the shelf life of fresh products^16^. In this work, the gas exchange of normal PE pouch (CK) was sufficient to maintain high O_2_ (13.37–20%) and low CO_2_ (0–2.5%) during storage. While that in the case of MP20 pouches, the content of O_2_ decreased, whereas the CO_2_ content increased during a transient period, and reached a steady state different from air atmosphere of approximately 2% O_2_ and 5% CO_2_ at day 2 (Table 1). This indicates that the two packaging meets the definition of MAP with different steady atmosphere of O_2_ and CO_2_, which might have different preservation effects on the mulberry leaf vegetable.

**Table 1:** The gas composition, soluble solids (TSS) and soluble protein of mulberry leaf vegetable in two packaging during storage. Each datum is the means and standard deviation (n = 3). *MP20* modified polyethylene packaging, *CK* normal polyethylene packaging.

	0 d	2 d	4 d	6 d	8 d
**O**_**2**_**content (%)**					
MP20	20 ± 0a	2.50 ± 0.08a	2.00 ± 0a	2.53 ± 0.08a	1.68 ± 0.35a
CK	20 ± 0a	13.38 ± 0.34b	15.27 ± 1.30b	14.73 ± 1.15b	16.35 ± 0.80b
**CO**_**2**_** content (%)**					
MP20	0 ± 0a	6.90 ± 0.12a	6.10 ± 0a	5.17 ± 0.05a	4.95 ± 0.07a
CK	0 ± 0a	2.50 ± 0.08b	2.00 ± 0b	2.53 ± 0.08b	2.10 ± 0b
**TSS (%)**					
MP20	10.55 ± 0.20a	10.42 ± 0.36a	11.65 ± 0.21a	10.95 ± 0.44a	11.03 ± 0.25a
CK	10.55 ± 0.20a	10.91 ± 0.39b	11.46 ± 0.36a	11.92 ± 0.67b	11.79 ± 0.65b
**Soluble protein (mg/g)**					
MP20	12.61 ± 0.69a	16.31 ± 0.29a	15.96 ± 0.68a	17.30 ± 0.48a	17.44 ± 0.33a
CK	12.61 ± 0.69a	16.48 ± 0.27a	16.53 ± 0.76b	17.32 ± 0.54a	18.31 ± 0.53b

Different letters (a, b) represent significant difference at *p* < 0.05 level with the same row between different packaging.

### Respiration rate

**Figure 1:**
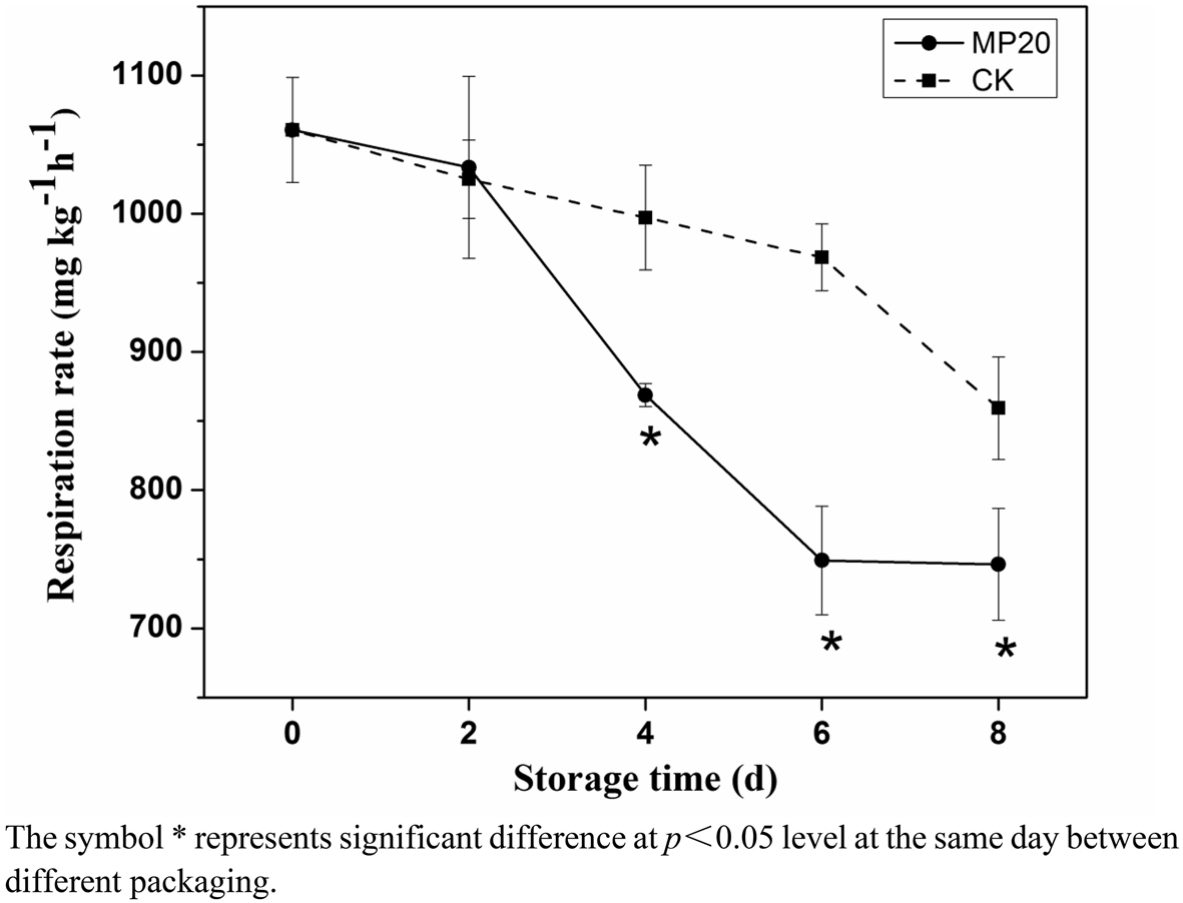
Effect of packaging on respiration rate of mulberry leaf vegetable during storage. Error bars indicate standard deviation. Asterisks indicate significant differences at the same storage time (*p* < 0.05).

The primary effect of MAP is to lower respiration rate of fresh produces, which ultimately reduces the rate of quality deterioration^17^. As expected, the respiration rate of mulberry leaf vegetable in MP20 was significantly lower than that in CK (Fig. 1). At days 4–8, the respiration rate of samples in CK was 997.21–859.37 mg CO_2_ kg^−1^ h^−1^, while that in MP20 was 868.80–746.33 mg CO_2_ kg^−1^ h^−1^, suggesting that MP20 packaging has benefits for inhibiting the respiration rate of mulberry leaf vegetable.

The lower respiration rate in MP20 may be related to the lower content of O_2_; a similar phenomenon was observed by Barrios, Lema and Lareo, who reported that a low level of O_2_ could down-regulate the respiration rate of strawberries^18^. Considering that respiration rate inhibition is beneficial for prolonging shelf life of fruits and vegetables^19^, it can be inferred that the quality of mulberry leaf vegetable is better when packaged with MP20 vs CK.

### Visual quality

**Figure 2:**
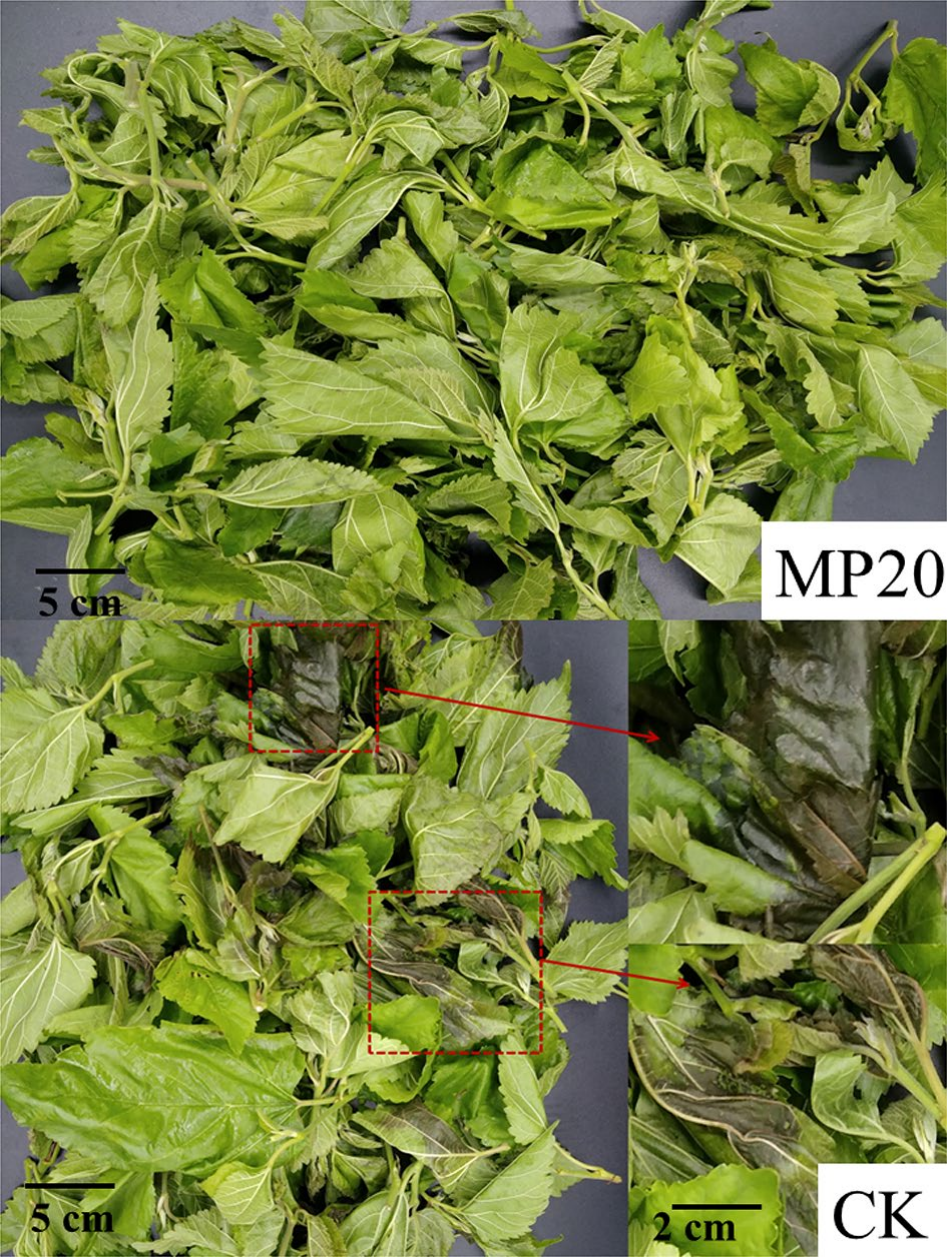
The visual quality of mulberry leaf vegetables in two packaging after storage 8 days at 4 °C. *MP20* modified polyethylene packaging, *CK* normal polyethylene packaging.

After 8 days storage, there was no obvious etiolation of mulberry leaf vegetable in both treatments and the water loss was minor (0.8% in MP20 and 1.3% in CK, results no shown). All the samples kept green, suggesting that low-temperature storage inhibits the yellowing of mulberry leaf vegetable for short time periods. However, there was moderate decay in CK, and some of the leaves turned brown (Fig. 2), suggesting that a packaging with larger gas permeability is not suitable for mulberry leaf vegetable storage under cold temperature.

### Total soluble solid content and total soluble protein

As shown in Table 1, the TSS content of mulberry leaf vegetable during storage was increased in both treatments, which was in agreement with the TSS changes of celery stored in air and in a controlled atmosphere. The authors suggested the TSS increased was related to a progressive metabolism of organic reserves to obtain the energy needed to maintain a higher metabolic activity, which could induce senescence of the product^20^. Moreover, the increase in CK was greater than that in MP20, suggesting MP20 packaging is effective in inhibiting the organic metabolism of mulberry leaf vegetable.

During storage, the soluble protein content of samples in both treatments increased significantly. The largest quantities of soluble protein in MP20 and CK were detected on day 8, that is, 17.44 ± 0.33 mg/g and 18.31 ± 0.53 mg/g (Table 1), respectively. A similar phenomenon was observed in the minimal processing of broccoli, where the authors suggested that the increase in protein content was related to the stress proteins that were induced in plants in response to various factors, such as temperature and oxygen^21^. Considering that the protein level in MP20 was lower than that in CK, and the water loss in both two treatments was negligible, we can reasonably infer that MP20 packaging may reduce stress response and metabolism of mulberry leaf vegetable after harvest, which may delay their quality deterioration.

### Total polyphenol and vitamin C content

**Figure 3:**
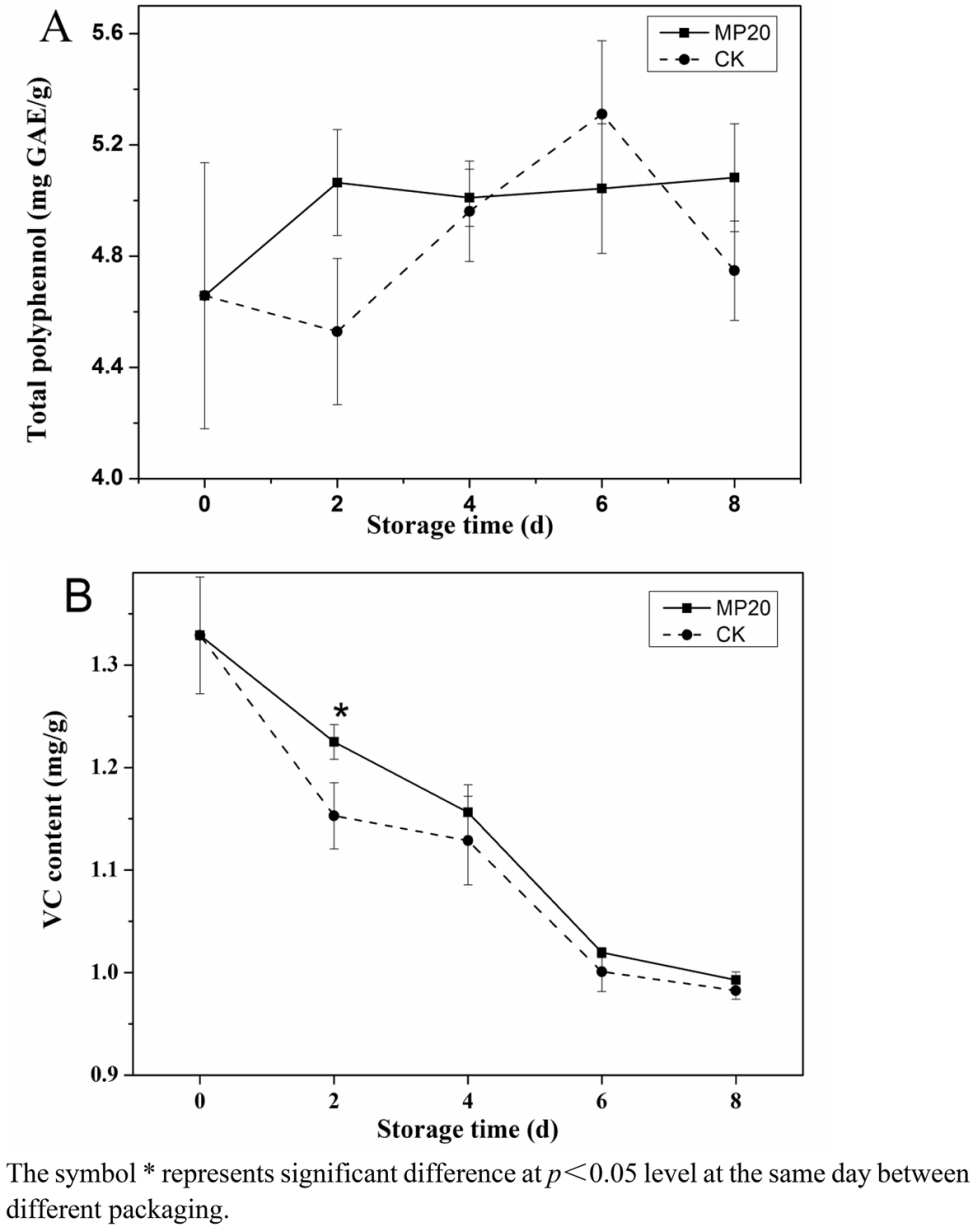
Effect of packaging on total polyphenol content (**A**) and vitamin C content (**B**) of mulberry leaf vegetable during storage. Error bars indicate standard deviation. Asterisks indicate significant differences at the same storage time (*p* < 0.05).

Polyphenol is an important nutrient and functional component of mulberry leaf vegetable, and has been shown to have antioxidant, anti-aging, anti-inflammatory and anti-obesity properties^1,2^. The phenols contents of mulberry leaves vary with variety, cultivation, maturation, and processing. The fresh mulberry leaf vegetable had high total polyphenol content of about 4.66 mg GAE/g just after harvest, which was higher than that reported by Ma et al.^22^. As expected, the total polyphenol content changes in mulberry leaf vegetable during storage were different between MP20 packaging and CK (Fig. 3A). In the former case, the content of total polyphenol increased gradually in first 2 days and then remained stable during the following storage days, while that in the latter increased significantly in first 6 days and then decreased notably at day 8 (Fig. 3A). A similar phenomenon was observed in cold-store blueberries. The authors suggested that the reduction in total polyphenol content was related to the polyphenol consumption in the browning reaction, where polyphenols were consumed as substrates of PPO^23^. The higher total polyphenol content remained in MP20 might be attributed to the lower respiration rate (Fig. 1), which inhibited the oxidative stress reaction of mulberry leaf vegetable during storage.

Vitamin C is an important nutrient in fruits and vegetables, and is easily degraded after harvest. As shown in Fig. 3B, the vitamin C content of mulberry leaf vegetable in both treatments was decreased gradually during storage, and the degradation rates in MP20 were lower than those in CK, indicating that vitamin C in the mulberry leaf vegetable had lower oxidization rates and lower loss of nutrients in MP20 packaging than in CK. Moreover, vitamin C is important for plant stress resistance because of its antioxidant activities. The higher vitamin C content remained in MP20 might reduce oxidative stress, thus kept the quality of mulberry leaf vegetable.

### Lignin content

**Figure 4:**
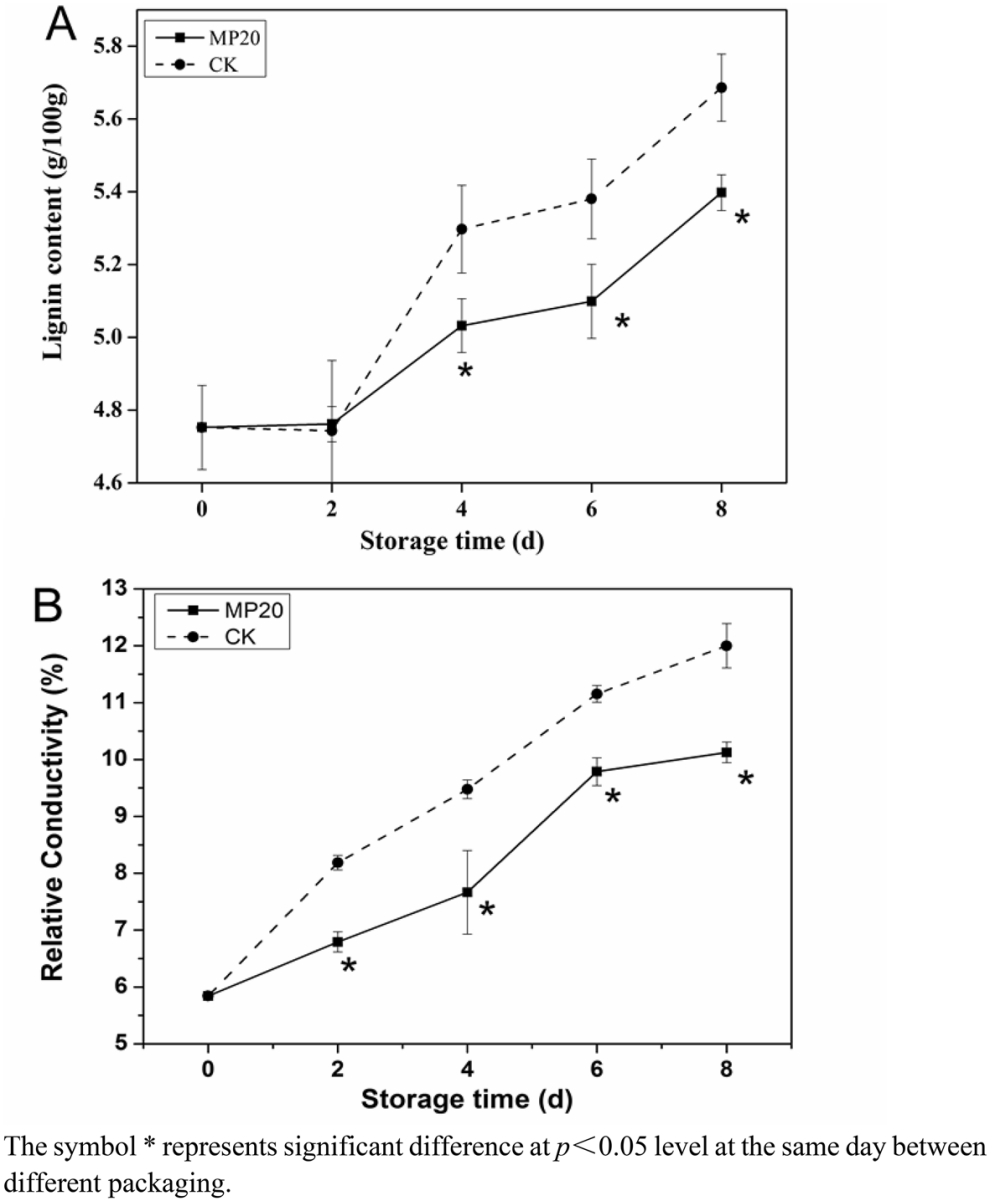
Effect of packaging on lignin content (**A**) and relative conductivity (**B**) of mulberry leaf vegetable during storage. Error bars indicate standard deviation. Asterisks indicate significant differences at the same storage time (*p* < 0.05).

Lignin accumulation in vegetables is related to their stress response to an inhospitable environment^24,25^. In our previous study, we found that mulberry leaf vegetable were prone to lignification after harvest. Lignin, the main component of secondary walls of plant cells, has a negative effect on the taste of vegetables^26,27^. As shown in Fig. 4 A, the lignin content of samples in both treatments increased gradually during storage, and the increasing rate in CK were larger than that in MP20 packaging (19.79% vs 13.38% at day 8), suggesting that MP20 packaging is more effective for inhibiting the lignin formation and maintaining the edible quality of mulberry leaf vegetable.

Polyphenols were generally regarded as precursors for lignin biosynthesis, and phenolic accumulation is important for lignification^28^. This might be the reason why the total phenols contents in CK increased significantly during the first 6 days storage (Fig. 3A). Moreover, MP20 packaging had a better influence on the maintenance of total phenols content during storage, which was in agreement with electrostatic atomized water particle treatment delay phenolic content reduction of asparagus. The authors suggested that the phenols content maintenance was related to the inhibition of conversion of phenols to lignin^29^. The high content of total phenols in MP20 maybe inhibit the activity of PPO and POD enzymes, and reduce the biosynthesis of lignin.

### Relative conductivity

Cell membrane damage is related to the senescence of vegetables after harvest, which is usually characterized by relative conductivity. During storage, the relative conductivity of mulberry leaf vegetable in both treatments increased gradually. However, the relative conductivity of samples in MP20 was significantly lower than that in CK, namely, 5.84–10.12% vs 5.84–12.00% (Fig. 4B), suggesting that the senescence of mulberry leaf vegetable was delayed by MP20 packaging.

### PPO and POD activities

**Figure 5:**
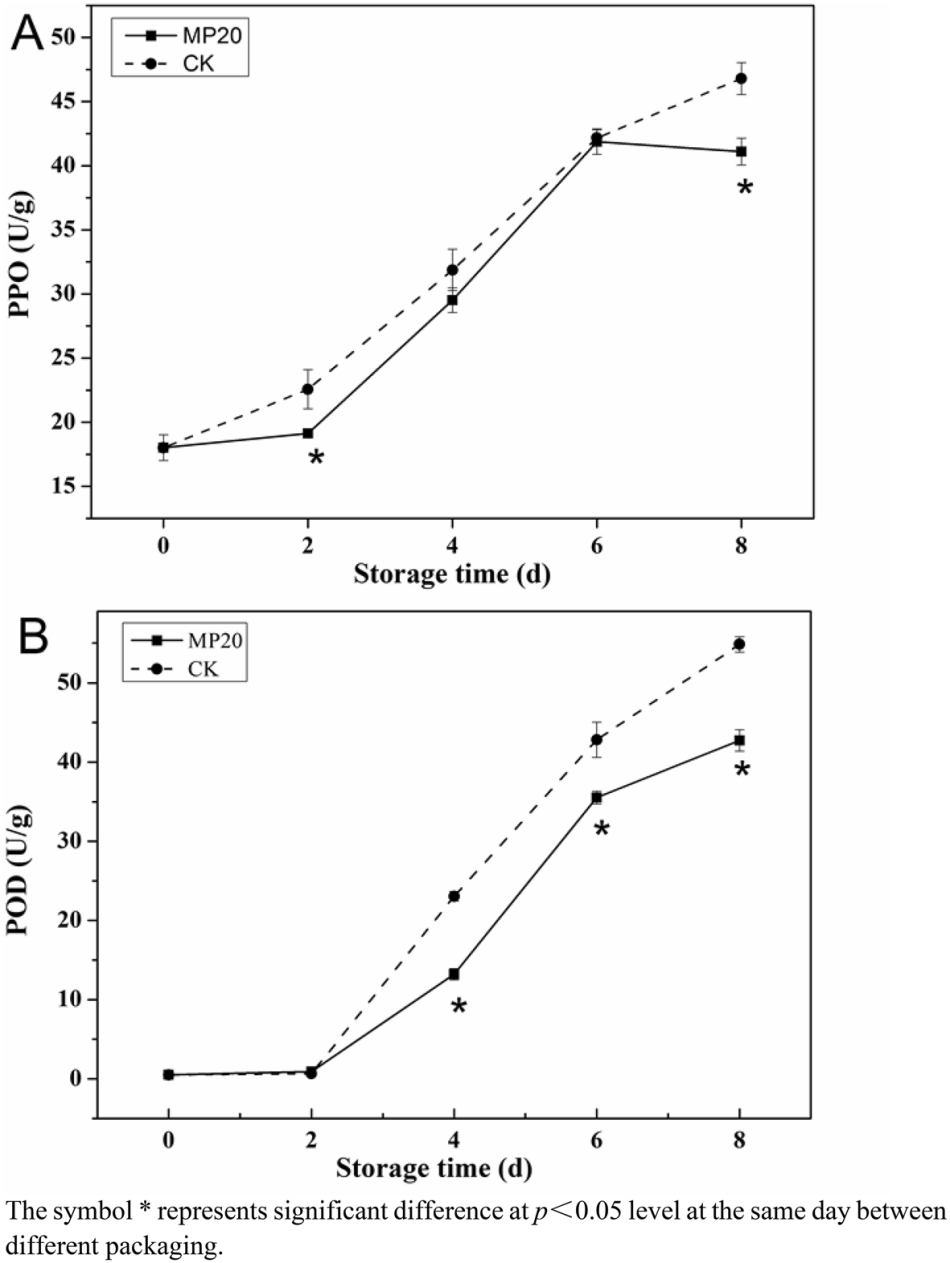
Effect of packaging on PPO activity (**A**) and POD activity (**B**) of mulberry leaf vegetable during storage. Error bars indicate standard deviation. Asterisks indicate significant differences at the same storage time (*p* < 0.05).

The positive correlation between PPO and lignification is debatable. It has been reported that lignin is positively correlated with PPO in harvested kiwifruit^30^, while PPO does not contribute to the lignification of bamboo shoots in MAP^31^. In this work, PPO activities in all samples gradually intensified during 8 days of storage, and MP20 packaged samples displayed lower PPO activities than those in CK (Fig. 5A). According to the Pearson correlation analysis, PPO activity was positively corrected with lignin content (r^2^ = 0.766, *p* < 0.01), indicating that PPO is closely related to the lignification of mulberry leaf vegetable. Moreover, there was a significant positive correlation between PPO activity and relative conductivity (r^2^ = 0.883, *p* < 0.05), suggesting that membrane damage would lead to PPO activity up-regulation. A similar phenomenon was observed in a previous study^32^. Increasing PPO activity could lead to the acceleration of polyphenols oxidation, which would promote the synthesis of lignin by participating in the oxidation of phenolic substances such as chlorogenic acid and coumaric acid^33^.

POD, a key antioxidant enzyme, is important for lignin synthesis in plant^34^. As shown in Fig. 5B, the POD activities of samples in MP20 packaging were lower than those in CK during storage. Moreover, the lignin content was closely related to the POD activity (r^2^ = 0.791, *p* < 0.01), suggesting that MP20 can retard the lignification by inhibiting POD activity. In summary, MP20 was a treatment with more effectively inhibited activities of phenolic metabolism-related enzymes, and thereby reduced phenols synthesizing into lignin, maintained high phenolic content and retarded lignification formation in fresh mulberry leaf vegetable.

To our knowledge, it was the first time to report the preservation technology to quality maintenance and lignification inhibition of fresh mulberry leaf vegetable after harvest. Mulberry leaves are prone to lignification due to their high respiration and metabolic processes. The lignin content was positively related to the firmness of the stem, and did negative effect on edible quality of leaf vegetable^27^. Wounding in handing stage could induce the increase of phenolic metabolism-related enzyme activities, and accelerate the synthesis of lignin. PPO and POD took part in the polymerization of lignin-based macromonomer and the accumulation of lignin polymers^35^. In the present work, the increase of TSS and soluble protein was suppressed as a result of stress response and metabolism of mulberry leaf vegetable reducing by MP20 packaging (Table 1). Moreover, the application of a suitable MAP (MP20 packaging) could better inhibit phenolic metabolism-related enzyme activities (PPO and POD) so as to reduce lignin biosynthesizing (Figs. 4, 5). Therefore, the MP20 packaging with moderate gas permeability is effective for nutrition maintenance and lignification inhibition of fresh mulberry leaf vegetable, thus MP20 packaging is a suitable kind of modified atmosphere packaging for mulberry leaf vegetable under cold storage.

## Conclusions

The effects of modified atmosphere packaging on the maintaining physiological quality and reducing lignification of fresh mulberry leaf vegetable during 8-d storage was studied in this research. Quality results revealed that the vitamin C content was higher, the total polyphenol content was more stable in MP20 packaging than that in CK. Furthermore, MP20 packaging alleviated lignin deposition by inhibiting cell membrane damage and suppressing phenolic metabolism-related enzyme activities. Generally, MP20 packaging is a suitable kind of modified atmosphere packaging for mulberry leaf vegetable preservation under cold storage. The molecular mechanism involved in lignin biosynthesis pathway needs to be further investigated.

## Data Availability

The datasets generated during and/or analysed during the current study are available from the corresponding author on reasonable request.
